# The Impact of Aerobic Exercise on Quality of Life in Women with Breast Cancer: A Randomized Controlled Trial

**Published:** 2016-07-31

**Authors:** Fatemeh Shobeiri, Seyedeh Zahra Masoumi, Azita Nikravesh, Rashid Heidari Moghadam, Manoochehr Karami

**Affiliations:** ^a^ Mother & Child Care Research Center, Hamadan University of Medical Sciences, Hamadan, Iran; ^b^ Department of Midwifery, School of Nursing and Midwifery, Hamadan University of Medical Sciences, Hamadan, Iran; ^c^ Department of Ergonomics, School of Public Health, Research Center for Health Sciences, Hamadan University of Medical Sciences, Hamadan, Iran; ^d^ Department of Epidemiology, School of Public Health, Social Determinants of Health Research Center, Hamadan University of Medical Sciences, Hamadan, Iran

**Keywords:** Exercise, Quality of Life, Breast Cancer, Women

## Abstract

**Background:** The women with breast cancer experience high rates of morbidity due to different treatments. The objective of this study was to evaluate the role of aerobic exercise in the quality of life (QoL) among women suffering from breast cancer in Hamadan, western Iran.

**Method:** Participants who had consummated the eligibility criteria were randomly assigned in exercise group (n=30) and control group (n=30). Written informed consent was obtained from all participants. The mean age was 42.70 ±9.6 and 43.50 ±8.60 yr old in exercise and control groups, respectively. The quality of life was assessed by two widely used standard questionnaires (EORTC QLQ-C30 and EORTC QLQ-BR23). The exercise group received supervised exercise 2 days per week for 10 weeks. Through two stages (before and after intervention) these groups were evaluated. Analyzing the data was performed by SPSS/20.0, using t-test, chi-squared and ANCOVA. *P*<0.05 was regarded as significant level.

**Results:** The global health status QoL, based on EORTC QLQ-C30, developed significantly in the exercise group (48.76±24.96 vs. 81.79±16.34) in comparison with the controls (47.75 ±15.73 vs. 52.88 ±14.51) (*P*<0.001). The exercise intervention was associated with substantial development in total score of functions and symptoms of QoL using EORTC QLQ-BR23 (*P*<0.001).

**Conclusions:** The statistically and clinically crucial developments were indicated in functions and symptoms of QoL in response to exercise in breast cancer women.

## Introduction

Breast cancer is known as the most generally diagnosed invasive cancer in women{Garcia, 2007 #1}^1^. According to the American Cancer Society, over 1.3 million women are diagnosed with breast cancer every year^1^. Iran, as a developing nation, is in medical transition from communicable to non-communicable diseases. Although cancer is the third cause of death in Asian countries, its mortality rate has increased during this decade^2^. Cancer is the third leading cause of death across the world, and the global burden has raised more than two fold over the past 30 year^2^. Regarding to the high mortality rate because of breast cancer in developing countries and the rising of its incidence, an epidemiologic evaluation with demographic trend analysis adjusting the shift in the age of presentation should be weighed for prevention of breast cancer^2,3^.

In Iran, breast cancer is first ranked among cancers diagnosed in women, comprising 24.4% of all malignancies with a crude incidence rate of 17.4 per 100,000, respectively^4^. The average age of Iranian women suffering from breast cancer is in the range of 47.1 - 48.8 yr, which is at least 10 yr less compared with the average age of those women living in developed countries^5,6^.

Fortunately, items such as early diagnosis, successful treatment, and modification of risk factors have raised survival rates of breast cancer in recent years. On the other hand, the treatments of breast cancer have yet adverse effects, which may negatively affect the quality of life (QoL) after the primary treatments. QoL issues for women with breast cancer include several factors such as pain, fear of relapse, fatigue, altered sense of femininity, feelings of diminished attractiveness, anxiety, and problems related to arm swelling^7^. Adverse physical and psychological side effects can be inherited due to cancer and its medications. Which contain a raising level of weakness and deficiencies in QoL, which can proceed for years after identification and have future influence on fatality^8,9^? Multiple parameters have effects on quality of life, which include an individual’s physical, functional, emotional and social well-being^10^. The evaluation of QoL in this population is claimed due to validated scales and questionnaires specifically designed for this cohort. Exercise is one of the potential approaches to overcome several problems such as cancer-related fatigue, loss of appetite, decrease in functional mobility^11^, and addresses depressive symptoms in other studies. In spite of this, previous research^12^ showed a positive relationship between the exercise interventions and fatigue in women with breast cancer. Therefore aerobic or combined modality training was conducted during chemotherapy to minimize fatigue in these women^12^. These authors showed trends for reducing the fatigue. There is another study on the impact of exercise on QoL in breast cancer women^13^. Predictors of preodic mastalgia are age, history of abortion and history of premenstrual syndrome, but it is inversely affected by the usage of hormonal contraceptive methods and exercise activity^14^. The literature report aerobic exercise in breast cancer women improved QoL outcome^15-17^. Thus, additional data is required to confirm the effect of aerobic exercise to elicit clinically relevant and sustained developments in QoL for women with breast cancer in Iran.

Residual dysfunction of the upper extremity related to surgical diagnostic and treatment methods and their side effects may be one of the contributors in reducing QoL in breast cancer. Breast cancer women report significant limitations in upper body strength in the months following their treatment the example of which is that many breast cancer survivors report shoulder pain and disability. From 9% to 28% of women had limitation of movement in arm and shoulder at 6 months after cancer treatment^18^. In addition to the mentioned reasons for aiming QoL, a unique range of musculoskeletal adaptations is recommended by aerobic exercise mainly important to help survivors of breast cancer; adaptations that are commonly not observed subsequent aerobic exercise.

In Iran, there have been very limited resources on the effect of aerobic exercise on quality of life in women with breast cancer. In this study, the effects of moderate-intensity aerobic exercise were evaluated on QoL and physical functioning in breast cancer women who had undergone surgery, chemotherapy or radiotherapy with or without hormone therapy usage. The hypotheses that would develop QoL were examined during 10 weeks of supervised exercise group.

## Methods

### Study Design and Subjects

A prospective, randomized controlled trial (RCT) was carried out on breast cancer survivors. The trial was registered with the Iranian registry of clinical trials (IRCT201412066888N5).

This RCT compared the outcomes of breast cancer patients assigned to a 10-week experimental treatment group (aerobic exercise) with those assigned to the control group. Outcome measures were gathered at week 0 and week 10. The samples were recruited from Shahid Beheshti Hospital and Mahdieh Therapy Center, Hamadan, western Iran from October 2014 to March 2015.

Sample size was estimated based on a previous study by Taleghani et al. ^19^. The level of significance was set at 5% (α=0.05), while the power of the study (1-β) was set at 80%. Thirty women enrolled in the each group.

This study was approved by Hamadan University of Medical Sciences, Hamadan, Iran (approval number: 4180). All procedures performed involving human participants were in accordance with the ethical standards of the institutional and/or national research committee and with the 1964 Helsinki Declaration protocol and its later amendments or comparable ethical standards.

Participants were randomly selected using a computer-generated list (www.randomization.com) stratified by age and current use of hormonal therapy. An investigator who had not been involved in testing or the delivery of the intervention prepared the randomization assignments. Participants who had the eligibility criteria were randomized to either the exercise group (n=30) or the control group (n=30).

Participants were pre-screened for participation, and those who were considered “moderate-risk” ^20^ received the approval of their physician prior to participation. Inclusion criteria encompassed a history of histological confirmed stage I–II breast cancer with no evidence of metastasis; 20 to 60 years of age, completed surgery and chemotherapy or radiotherapy with or without current use of hormonal therapy (e.g. tamoxifen and aromatase inhibitors), do not use other methods of alternative medicine (massage, rehabilitation, etc.), lack of regular exercise, and lack of mobility problem. Exclusion criteria included increased severity of breast cancer, death, and metastasis.

### Intervention

Women determined to the intervention group attended a supervised group exercise program two times per week for approximately 40-60 min per session for 10 weeks. The training sessions designed for ten weeks consisted of group training with multiple participants per trainer (8–10) exercising concurrently in the gym.

The American College of Sport Medicine (ACSM) has F.I.T.T. guidelines for strength training. Exercise program was planned based on the F.I.T.T. principles. This acronym stands for Frequency, Intensity, Time, and Type.

The exercise program included a warm-up period, followed by moderate intensity of aerobic exercises, and completed by a cool-down period. Warm-up period entailed 5-10 min of slow walking and moderate stretching physical activity. The duration of aerobic exercise was initially 15 min and was distributed equally among the three exercise modalities (moderate walking, stretching physical activity, and specific movements of arms and shoulders). The sessions ended with 5 min of cool-down exercises, which involved walking slowly. Following standard guidelines for exercise prescriptions ^20^, warm-up and cool-down period remained the same for 10 weeks until the aerobic exercise period increased by 2% in each week, resulting in 35 min in week 10. The participants exercise intensity was prescribed based on the Karvonen formula ^7, ^^21^.

Heart rate reserve (HRR) was employed to calculate training zones based on both maximum and resting heart rate, using a range of 50%-75%. The maximum heart rate (HR_max_) was computed via the formula: HR max=220–Age. The heart rate was monitored by the participants and the supervisor during exercise through Polar FT4 heart rate monitor. During the 10-week exercise program, exercise intensity increased from 50% to 75% HRR, and exercise duration rose from 25 to 45 min per session both in almost linear fashion. Women in the control group were told to maintain their sedentary lifestyle for 10 weeks. The control group was not advised to change habitual activity levels, and both groups continued to receive usual medical care^20^.

### Measurements

The survey included questions on demographic and medical variables. Demographic data (name, age, reproductive history, occupational condition, education level, etc.) were collected by interviewing the participants. Medical data (the number of days after the diagnosis and the end of treatments, cancer stage, medication, type of treatment, and type of surgery) were obtained from medical records.

The primary outcome measure was overall QoL, as measured by the European Organization for Research and Treatment of Cancer Core Cancer Quality of Life Questionnaire (EORTC QLQ-C30) and the European Organization for Research and Treatment of Cancer (EORTC QLQ-BR23) scales ^22^. The EORTC QLQ-C30 is a 30-item scale that measures quality of life in cancer patients who receive scores for functional scale (15 items), symptom scale (13 items), and global health Status (2 items). Each item is rated on a scale from 0 (not at all) to 4 (Very much). High scores for functional and global health scales indicate a good quality of life, while high scores in symptom scale represent a high level of problems^22^.

The EORTC QLQ-BR23, as a tumor specific tool, consists of 23 items. These items pertain to the dimension body image (4 items), sexuality (3 items), future perspective (1 item), and side effect related to different treatment modalities such as surgery, chemotherapy or radiotherapy (15 items). Questions also address sexual interest, sexual activity, and sexual satisfaction. Each item is graded on a scale from 0 (not at all) to 4 (Very much). With respect to functional scale, higher scores represent higher levels of functioning, whereas higher scores in symptom-orientated scales correspond to higher levels of symptoms^22^.

The EORTC QLQ-C30 and EORTC QLQ-BR23 are highly reliable with test–retest correlations of 0.71–0.95 ^23^. Body weight and height were measured on a calibrated scale. Body mass index was calculated, too. Finally, the outcomes were assessed at baseline and post intervention.

### Statistical analysis

Primary analysis was executed via intention-to-treat, which included all participants, regardless of dropout or level of adherence. Missing data in week 10 were imputed; using the last observation carried forward method. The data were presented as the mean and SD. Baseline characteristics were compared between groups using independent *t*-tests for continuous variables and chi-square tests for categorical variables. Primary analysis compared changes in QLQ-C30 and QLQ BR-23 via ANCOVA, where the final score was the dependent variable, and the baseline value of the same variable was the covariate. *P* value less than 0.05 was considered indicative of statistical significance. Data processing and statistical analysis were performed, using SPSS version 20.0 (Chicago, IL, USA).

## Results

**Figure 1:**
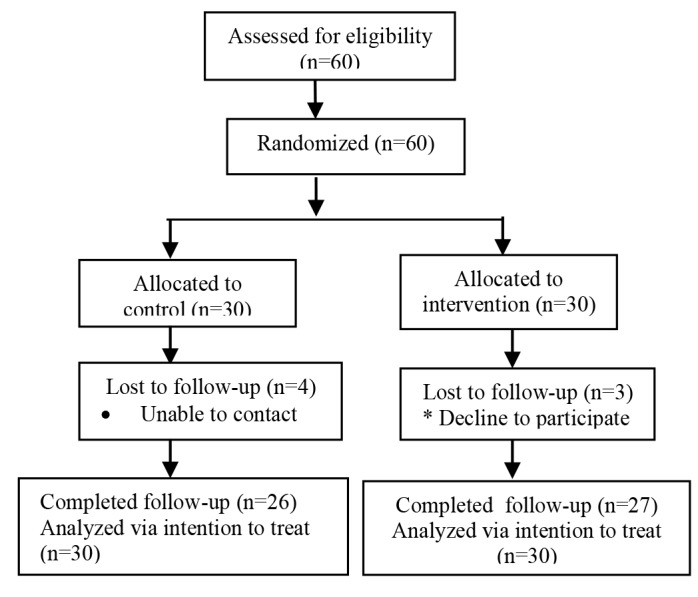
Flow of participants through the trial

Sixty women enrolled in the study. They were randomly assigned in the exercise group (n=30) and the control group (n=30). Seven women were excluded due to failure to meet inclusion criteria or declining interest. The major reason for not meeting the criteria was that of a current cancer diagnosis. Fifty-three women enrolled in the exercise (n=27) and the control group (n=26). Participant flow through the trial is presented in Figure 1.

Table 1 demonstrates demographic and medical characteristics of participants. Totally, the two groups were similar at baseline. No significant differences were found between the groups in terms of age, BMI, education, occupation, and parity. Mean age was 42.70±9.00 and 43.50±8.60 and mean menarche age was 12.00±1.80 and 12.53 ±1.50 yr in exercise and control groups, respectively.

**Table 1:** Baseline characteristics and clinical data of the study population

**Characteristics**	**Control n=26 (30)**	**Exercise n=27 (30)**	
**Continuous variables**	**Mean (SD)**	**Mean (SD)**	***P *** **value**
Age (yr)	43.50 (8.60)	42.70 (9.60)	0.810
Menarche age (yr)	12.53 (1.50)	12.00 (1.80)	0.240
Marriage age (yr)	17.34 (6.80)	18.22 (7.48)	0.650
Age at first delivery (yr)	19.84 (7.90)	19.33 (8.78)	0.820
Number of parity	2.53 (1.30)	2.55 (1.60)	0.960
Breast feeding (month)	33.42 (21.10)	33.7 (27.30)	0.960
Body mass index (kg/m^2^)	27.21 (4.40)	27.68 (4.90)	0.710
**Categorical variables**	**Number (%)**	**Number (%)**	***P *** **value**
Educational level			0.790
Illiterate	3 (11.50)	3 (11.10)	
literate	23 (88.50)	24 (88.90)	
Working status			0.260
Employed (%)	6 (23.10)	10 (37.00)	
Unemployed	20 (76.90)	17 (63.00)	
Family history of breast cancer (%)		1.000
Yes	2 (7.70)	3 (11.10)	
No	24 (92.30)	24 (88.90)	
History of breast surgery			0.680
Mastectomy	13 (50.00)	12 (44.40)	
Lumpectomy	13 (50.00)	15 (55.60)	
History of radiation therapy		0.490
Yes	13 (50.00)	11 (40.70)	
No	13 (50.00)	16 (59.30)	
History of breast chemotherapy		0.610
Yes	24 (92.30)	26 (96.30)	
No	2 (7.70)	1 (3.70)	
Hormone replacement therapy		0.490
Yes	11 (42.30)	15 (55.60)	
No	15 (57.70)	12 (44.40)	

The outcomes of QoL measures are reported in Table 2. The baseline values for the global health status QoL measures were not different between the two groups. Kolmogorov-Smirnov test demonstrated no significant differences between the groups (*P*> 0.05).

**Table 2:** Comparative of measurements of quality of life (QLQ-C30) between groups

**Variables**	**Baseline** **(mean ±SD)**	**After 10 weeks** **(mean ±SD)**	***P*** ** value**
**Global health status/QoL**			0.001
Exercise	48.76 ±24.96	81.79 ±16.34	
Control	47.75 ±15.73	52.88 ±14.51	
**Functional scales**			
Physical functioning			0.001
Exercise	68.39 ±16.59	87.65 ±8.41	
Control	73.80 ±11.14	78.71 ±8.84	
Role functioning			0.001
Exercise	65.43 ±23.98	94.44 ±8.00	
Control	83.97 ±15.29	91.02 ±10.78	
Emotional functioning			0.001
Exercise	45.98 ±30.51	81.48 ±15.56	
Control	59.29 ±21.25	63.78 ±19.42	
Cognitive functioning			0.001
Exercise	71.60 ±26.88	91.35 ±14.14	
Control	85.89 ±19.25	91.66 ±11.78	
Social functioning			0.002
Exercise	63.58 ±29.24	83.95 ±14.96	
Control	69.87 ±23.57	80.76 ±18.06	
**Symptom scales/items**			
Fatigue			0.001
Exercise	44.44 ±24.45	18.51 ±14.45	
Control	43.16 ±18.94	37.60 ±11.79	
Nausea and vomiting			0.001
Exercise	37.25 ±11.07	27.16 ±24.52	
Control	19.23 ±17.44	31.41 ±22.27	
Pain			0.001
Exercise	44.44 ±24.01	16.66 ±16.01	
Control	41.02 ±20.12	31.41 ±14.39	
Dyspnea			0.001
Exercise	39.39 ±13.48	33.33 ±0.00	
Control	33.33 ±0.00	33.33 ±0.00	
Insomnia			0.040
Exercise	43.13 ±15.65	35.70 ±8.90	
Control	32.05 ±30.52	24.35 ±24.14	
Appetite loss			0.050
Exercise	37.03 ±29.71	22.22 ±18.49	
Control	41.02 ±21.72	35.89 ±18.60	
Constipation			0.001
Exercise	40.47 ±14.19	38.46 ±12.51	
Control	45.24 ±16.57	47.05 ±16.90	
Diarrhea			0.100
Exercise	26.74 ±17.28	33.33 ±0.00	
Control	47.61 ±17.82	33.33 ±0.00	
Financial difficulties			0.230
Exercise	55.55 ±34.59	65.43 ±36.37	
Control	48.71 ±23.53	67.94 ±33.40	

This table also summarizes the outcome variables evaluated at the baseline and 10-week post intervention for the participants in exercise and control groups separately. The global health status QOL, based on EORTC QLQ-C30, improved significantly in the exercise group (48.76 ±24.96 vs. 81.79 ±16.34) compared to controls (47.75±15.73 vs. 52.88±14.51) (*P*<0.001) and increased 33.03 for the exercise group and diminished –5.13 for the control group (*P*<0.001). Analysis of the pre- and post-intervention demonstrated a considerable development for functional scales: physical functioning, role functioning, emotional functioning, cognitive functioning (*P*<0.001), and social functioning (*P*=0.002), and for symptom scales: fatigue, nausea and vomiting, pain, dyspnoea, costipation (*P*<0.001), and insomnia (*P*=0.040). However, there was not any relationship in appetite loss (*P*=0.050), diarrhea (*P*=0.100), and financial difficulties (*P*=0.230) in both of the exercise and control groups.

There were not significant relationships at baseline between quality of life as evaluated by EORTC QLQ-BR23. The exercise intervention was related to a significant improvement in function: body image (*P*<0.001), sexual function (*P*=0.008), sexual pleasure (*P*=0.030), future prospect (*P*<0.001), and total score of functions (*P*<0.001). It also showed significant improve in symptoms: side effects of treatment, breast symptoms, arm symptoms, and worry about hair loss (*P*<0.001) (Table 3).

**Table 3:** Comparative of measurements of quality of life (QLQ BR-23) between groups

**Variables**	**Baseline** **(mean ±SD)**	**After 10 weeks** **(mean ±SD)**	***P*** ** value**
**Components of function**			
Body image			0.001
Exercise	58.64±27.78	87.3 ±14.85	
Control	64.10±25.36	73.39 ±17.93	
Sexual function			0.008
Exercise	88.88 ±28.11	80.24 ±31.70	
Control	89.10 ±24.90	80.76 ±27.76	
Sexual pleasure			0.030
Exercise	83.95 ±35.04	76.54 ±36.76	
Control	87.17 ±28.40	76.92 ±29.46	
Future prospect‏			0.001
Exercise	62.50 ±27.80	56.79 ±28.96	
Control	26.92 ±23.13	42.30 ±34.71	
Total score of functions			0.001
Exercise	65.50 ±6.46	27.16 ±26.61	
Control	71.20 ±4.80	78.37 ±10.56	
**Components of symptoms**			
Side effect of treatment			0.001
Exercise	41.26 ±20.15	17.63 ±13.12	
Control	34.98 ±15.35	25.09 ±14.20	
Breast symptoms			0.001
Exercise	25.61 ±18.62	30.70 ±5.55	
Control	13.80 ±5.40	24.67 ±13.43	
Arm symptoms			0.001
Exercise	42.38 ±23.47	12.12 ±10.69	
Control	23.07 ±21.29	25.64 ±17.43	
Worry of hair loss			0.001
Exercise	46.91 ±38.40	19.07 ±13.58	
Control	41.02 ±35.66	51.00 ±17.50	
Total score of symptoms			0.001
Exercise	37.69 ±17.21	17.21 ±12.75	
Control	26.49 ±12.18	24.87 ±10.07	

## Discussion

The survivors from cancer experience high rates of morbidity due to different treatments. Exercise has several benefits in improving treatment outcomes and quality of life. In India, a broad range of the benefits of exercise-based interventions was found^24^. Iran has a high prevalence of breast cancer, and there are a few researches about exercise interventions for cancer survivors in Iran. Our results established the hypothesis that 10 weeks of aerobic exercise program significantly modify the total of QoL and physical functioning in the survivors of breast cancer. The findings of the present research are similar to those of the previously published studies for the benefit of the aerobic exercise in the total aspects of QoL^7,25^. In this study with the exercise group, after 10 weeks, a noticeable development was determined based on QLQ-C30: global health status/QoL in comparison with primary values (change of 33.03 points), physical function (change of 19.26 points), role function (change of 29.01 points), emotional function (change of 35.50 points), cognitive function (change of 19.75 points), and social function (change of 20.37 points). Based on QLQ BR-23, the following results were obtained: body image (change of 28.66 points), sexual function (change of -8.64 points), sexual pleasure (change of -7.41 points), future prospect (change of 5.71 points), and components of symptoms (side effects of treatment (change of 23.63 points), breast symptoms (change of 0.61 points), arm symptoms (change of 24.33 points) and worry about hair loss (change of 10.91 points) (*P* < 0.05), supported by other studies ^25,26^ that were done on similar exercise intervention, demonstrating significantly higher levels of physical functioning as well as relating higher QoL scores compared to the control group. While, there were no differences established according to arm lymphedema or quality of life ^27^.

There has been a promotion in the level of health and educational activities related to performing breast self-exam in Iran ^28^. In a longitudinal study women with breast cancer noticed benefit from their cancer treatment in long-term ^29^. In other studies conducted the psychological distress-anxiety and depression investigation on women with breast cancer, established to be common among the breast cancer women even years after the disease diagnosis and treatment. Moreover, psychological parameters were recognized for predicting subsequent QoL ^30,31^.

Concerning health-related quality of life after the exercise period, the main finding of the present study showed that many functions and symptoms had decreased or vanished based on QLQ–C30. While according to QLQ Br-23, all components of function (body image, sexual function, sexual pleasure, and future prospect) and components of symptoms (side effect of treatment, breast symptoms, arm symptoms, and worry about hair loss) had decreased or disappeared. These results are in accordance with the results of another research^32^.

A similar randomized trial conducted on Australian women reported that the experimental group received supervised resistance training of 3 days per week for 16 weeks. Feeling the tiredness and QoL improved extensively in the experimental compared to the control^26^.

In a study that considers the effects of exercise on QOL of the patients with breast cancer undergoing chemotherapy reported that exercise intervention promoted patients’ QOL. A significant reduction was observed of the average score of spiritual health in the control group, while this average score was considerably enhanced in the test group (*P*=0.004). Generally, the total mean score of the quality of life showed no significant difference before and after intervention.

Moreover, the quality of life outcome improved in another study^19^. In the present study, the statistically and clinically crucial developments were indicated in functions and symptoms of QoL in response to exercise in breast cancer women. The results reflect the impact of aerobic exercise advice on the promotion of quality of life in breast cancer women.

The limitation of this study include lack of sample size and lack of cooperation by some patients due to cultural, physical, emotional problems.

The breast cancer treatment usually consists of surgery besides radiotherapy, chemotherapy, and hormonal treatment to diminish the risk of relapse. The cancer therapies could have substantial impacts on women quality of life.

## Conclusions

Exercise plays an effective and safe role in all parameters and important developments of global QoL in breast cancer women. Research involving exercise-based rehabilitation interventions is limited in Iran. With the growing burden of cancer in this country, there is an immediate demand for research on exercise-based interventions for breast cancer women in Iran.

## Acknowledgments

The authors would like to thank the Hamadan University of Medical Sciences in Iran for their valuable support and participation.

## Conflict of interest statement

The authors declare that they have no competing interests.

## Highlights

- Aerobic exercise in women with breast cancer leads to improvement in body image, sexual function and sexual pleasure.
- Aerobic exercise in women with breast cancer after 10 weeks decreases the side effects of treatment.
- Exercise plays an important role in quality of life among women with breast cancer.
